# Short Video Addiction Risk Transitions Among University Students: A Three-Wave Latent Transition Analysis of Unintentional Procrastination and Self-Concept Clarity

**DOI:** 10.3390/bs16101724

**Published:** 2026-09-22

**Authors:** Zh-Yeng Chong, Zhihui Zhang, Xinqi Chen, Wei Xu

**Affiliations:** Beijing Key Laboratory of Consciousness Brain Computer Interface and Neuromodulation, National Demonstration Center for Experimental Psychology Education (Beijing Normal University), Faculty of Psychology, Beijing Normal University, Beijing 100875, China; 202339061001@mail.bnu.edu.cn (Z.-Y.C.); 202338039111@mail.bnu.edu.cn (Z.Z.); chenxq@mail.bnu.edu.cn (X.C.)

**Keywords:** short video addiction, latent transition analysis, unintentional procrastination, self-concept clarity, university students, risk profiles

## Abstract

This study examined changes in short video addiction (SVA) among university students. Three-wave longitudinal data were used to identify relative SVA profiles, estimate transitions among these profiles, and examine the prospective associations of unintentional procrastination and self-concept clarity with subsequent profile membership. A total of 2145 students completed at least one assessment. The primary latent transition analysis included 1135 students with valid SVA data at two or more waves, and the predictor analyses included 679 three-wave completers. Three ordered profiles characterized by lower, moderate, and higher SVA were identified. These profiles represent relative latent classes within the present sample and are not diagnostic categories. Profile membership showed both stability and movement across adjacent waves. In the classification error-adjusted predictor analyses, higher unintentional procrastination was associated with lower odds of membership in the lower profile relative to the higher profile among students initially in the moderate profile. Higher self-concept clarity was associated with greater odds of maintaining lower or moderate profile membership relative to membership in the higher profile. These findings indicate that relative SVA profile membership can change over time and that unintentional procrastination and self-concept clarity show different prospective associations with subsequent profile membership.

## 1. Introduction

Short video addiction (SVA) has become a growing concern among university students, with recent university student studies reporting prevalence estimates of approximately 20.1% to 31.3% (X. Li et al., 2021; Mohd Nor Shokri et al., 2025; Tian et al., 2025). SVA can be situated within the broader literature on problematic digital media use (Brand et al., 2019; Qu et al., 2024). It shares features with other problematic online behaviors, including impaired control, persistent use despite negative consequences, and interference with everyday functioning (Brand et al., 2019; Mao et al., 2022; Qin et al., 2019). Short video platforms provide brief and highly engaging content, often delivered through personalized recommendation feeds (Xiong et al., 2024). Personalized short video content has also been associated with neural activity involved in attention and reward processing (Su et al., 2021). Among university students, higher SVA has been associated with greater academic procrastination (Xie et al., 2023). SVA among university students may therefore be reflected in difficulty limiting or controlling short video use when viewing begins to interfere with academic tasks, sleep routines, and other planned activities (Qin et al., 2019; Xie et al., 2023; Zhao & Kou, 2024).

Existing SVA studies have commonly used questionnaire measures, such as the Short Video Addiction Scale for College Students (Qin et al., 2019), the Problematic Short Video Media Use Scale (Mao et al., 2022), the TikTok Addiction Scale (Galanis et al., 2024), and adapted short video addiction scales (Ye et al., 2023), to compute total or mean severity scores. This approach is useful for examining whether higher SVA is related to poorer psychological, sleep, or academic outcomes (L. Li et al., 2024; Xie et al., 2023; Zhao & Kou, 2024). However, severity scores mainly indicate how high or low students’ SVA symptoms are on average (Mao et al., 2022; Qin et al., 2019). They provide less information about whether students can be meaningfully grouped into distinct risk patterns (Ding et al., 2024; L. Li et al., 2024; Pan et al., 2024; S. Wang & Liu, 2026). Consequently, an important unresolved question is whether comparable relative SVA profiles can be identified across repeated assessments and whether students remain in the same profiles or move to profiles characterized by lower or higher SVA levels over time. The present study addresses this question by using three-wave latent transition analysis to examine the comparability and movement of relative SVA profiles over time.

Recent profiling studies have shown that SVA is heterogeneous among university students, although the profiles identified have varied across studies. Pan et al. (2024) identified non-addiction, low-addiction, and high-addiction profiles, suggesting an ordered pattern of SVA severity. L. Li et al. (2024) identified five profiles, some of which were characterized by different patterns of withdrawal, escape, and loss of control. Other studies examined psychological characteristics associated with SVA profiles. Ding et al. (2024) identified three SVA profiles and found that the profiles differed in subjective well-being, social support, core self-evaluation, and extraversion. S. Wang and Liu (2026), in contrast, identified four profiles based on the joint patterning of SVA and emotion dysregulation. Because these studies used different indicators, their profile solutions are not directly comparable. Nevertheless, they consistently demonstrate that university students differ in their patterns of SVA and related psychological functioning. A central question remains unresolved because these studies assessed profiles at only one time point. It is therefore unclear whether the identified profiles represent relatively stable risk groups or temporary risk states that students may enter or leave over time.

Evidence from the broader literature on problematic digital media use suggests that risk profiles can change over time. G. Li (2023) identified non-addiction, low-addiction, and high-addiction internet use profiles across three waves and found that individuals in the low-addiction profile could subsequently transition either to the non-addiction profile or to the high-addiction profile. Hirota et al. (2021) also documented stability and change in Internet addiction status over two years, including transitions from non-addiction to addiction. These studies show that profiles of problematic digital media use may represent changing risk states. However, it remains unclear whether comparable patterns of stability and movement toward lower or higher SVA profiles occur among university students.

Within SVA research, longitudinal studies have examined associations between SVA and psychological functioning across waves using several cross-lagged approaches. Using a conventional cross-lagged panel model, Zhang et al. (2024) found that earlier short video overuse was associated with higher subsequent depressive symptoms, whereas earlier depression was not associated with subsequent short video overuse. This study clarified the longitudinal ordering of the association between the two constructs but represented SVA as a single continuous score. Qu et al. (2024) examined the association between SVA and depression at the symptom level using cross-lagged panel network analysis. They reported prominent cross-wave associations involving tolerance and anhedonia and identified conflict and sad mood as bridge symptoms connecting SVA and depression. This analysis provided symptom-level evidence of prospective associations across waves, but it did not examine whether students belonged to different SVA risk profiles or moved between those profiles over time. Using a random-intercept cross-lagged panel model, Zhang et al. (2025) separated stable between-person differences from within-person fluctuations and found that within-person elevations in self-concept clarity were associated with lower subsequent SVA, whereas SVA was not associated with subsequent self-concept clarity.

Taken together, the profiling and longitudinal literature address complementary aspects of SVA. Profiling studies identify heterogeneity among students but provide only a cross-sectional snapshot. Longitudinal studies examine associations across waves but represent SVA through continuous scores or individual symptoms. Evidence from broader forms of problematic digital media use indicates that movement between risk profiles is possible, but corresponding evidence for SVA remains limited. The present study connects these lines of research by using latent transition analysis to identify comparable relative SVA risk profiles across three waves and estimate transitions among them.

A goal-directed self-regulation perspective offers a theoretical framework for considering how characteristics related to self-regulation may be associated with movement among SVA profiles. Self-regulation involves guiding one’s thoughts, emotions, and behaviors in accordance with goals and standards, especially when immediate rewards conflict with longer term outcomes (Carver & Scheier, 1998). Core symptoms of SVA, such as loss of control, continued use despite negative consequences, and difficulty stopping, reflect difficulties in regulating short-term digital rewards in relation to longer-term goals (Mao et al., 2022; Qin et al., 2019). For university students, this conflict may appear in everyday situations, such as intending to study, sleep, or complete tasks but continuing to watch short videos instead (Xie et al., 2023; Ye et al., 2023). From this perspective, movement among relative SVA profiles may be associated with both the clarity of students’ goals and self-standards and their ability to translate intentions into action. Movement from a moderate or higher SVA profile to a lower profile may particularly involve implementing intentions to reduce an established pattern of use. Avoiding movement into a higher SVA profile may particularly involve maintaining goals and behavioral standards before problematic use becomes established. This distinction concerns the self-regulatory demand expected to be most relevant to each transition and does not assume that either characteristic operates exclusively in one direction. It is grounded in established work showing that self-concept clarity reflects clear and stable self-beliefs, whereas procrastination reflects self-regulatory difficulty in carrying out intended actions (Campbell et al., 1996; Fernie et al., 2017; Steel, 2007). Recent evidence has also associated lower self-concept clarity with weaker self-control and reduced global self-continuity (Jiang et al., 2023). Building on this evidence, the present study examined self-concept clarity and unintentional procrastination as two characteristics related to self-regulation that may show different prospective associations with SVA risk transitions.

Unintentional procrastination refers to delaying intended actions despite wanting to complete them, reflecting difficulty translating intentions into action (Fernie et al., 2017). Procrastination has commonly been conceptualized as a failure of self-regulation because it involves delaying an intended course of action despite potential negative consequences (Steel, 2007). In the context of short video use, this tendency may be especially relevant for students in the moderate or higher SVA profiles. Moving to a lower SVA profile may involve recognizing a need to change short video use and carrying out intentions to stop watching, reduce use, or return to academic tasks. Students with higher unintentional procrastination may experience greater difficulty implementing these intentions when short videos provide immediate and easily accessible rewards. Unintentional procrastination may therefore be associated with a lower likelihood of subsequent membership in a lower SVA profile because it reflects difficulty carrying out an intended change in short video use. Existing SVA studies have reported associations between short video addiction and academic or general procrastination using cross-sectional, mediation, or moderated mediation models (Xie et al., 2023; Zhao & Kou, 2024). However, these studies have primarily examined associations with SVA severity or related outcomes. It remains unclear whether baseline unintentional procrastination is prospectively associated with movement from moderate or higher SVA profiles to a lower profile.

Self-concept clarity refers to the extent to which individuals’ self-beliefs are clearly defined, internally consistent, and stable over time (Campbell et al., 1996). From a goal-directed self-regulation perspective, a clear and stable self-concept may provide internal standards relevant to behavioral guidance and goal pursuit. This function may be particularly relevant to maintaining lower or moderate SVA profile membership. For these students, avoiding escalation may involve maintaining limits on short video use before impaired control becomes more established. Clear and stable self-beliefs may provide standards for evaluating immediate digital rewards in relation to longer term goals and behavioral boundaries (Campbell et al., 1996; Jiang et al., 2023). Consistent with this view, recent longitudinal evidence indicated that higher self-concept clarity was prospectively associated with lower subsequent SVA severity (Zhang et al., 2025). Lower self-concept clarity may therefore be associated with a greater likelihood of movement toward a higher SVA profile. Lower self-concept clarity has been associated with Internet addiction and related cognitive difficulties (Y. Wang et al., 2022). However, existing studies have primarily examined continuous addiction severity or cross-lagged associations. The prospective association between self-concept clarity and movement into a higher SVA profile remains unclear.

Taken together, existing research has identified cross-sectional heterogeneity in SVA and longitudinal associations between SVA and psychological functioning. However, it remains unclear whether students move among relative SVA profiles over time and whether subsequent profile membership shows different prospective associations with characteristics related to self-regulation. The present study used three-wave longitudinal data and latent transition analysis to identify comparable relative SVA profiles, estimate transitions among them, and examine the prospective associations of unintentional procrastination and self-concept clarity with T3 profile membership within each T1 latent profile.

The study addressed the following research questions and hypotheses. Research Question 1 (RQ) examined whether comparable relative SVA profiles could be identified among university students across three waves. Research Question 2 (RQ2) examined patterns of stability and movement among these profiles over time. Hypothesis 1 (H1) proposed that, among students initially in the moderate or higher SVA profiles, higher unintentional procrastination would be associated with lower odds of subsequent membership in a lower SVA profile. Hypothesis 2 (H2) proposed that, among students initially in the lower or moderate SVA profiles, higher self-concept clarity would be associated with lower odds of subsequent membership in a higher SVA profile.

## 2. Method

### 2.1. Participants

A total of 2145 university students completed at least one wave of a three-wave longitudinal survey. Valid SVA data were available for 1592 students at T1, 1352 students at T2, and 1015 students at T3. The primary LTA sample comprised 1135 students who provided valid SVA data at two or more waves. Participants who completed only one wave were retained in the merged dataset but were not included in the transition analysis because they could not contribute information on change in profile membership over time. The primary T1 to T3 predictor analyses were conducted among 679 students who completed all three waves and had valid baseline unintentional procrastination, self-concept clarity, and gender data. Of these students, the mean age was 19.40 years (SD = 0.61, range = 18–21) and 88.2% were female. A participant flow diagram is presented in Figure S1 in the Supplementary Materials.

Among the 1592 participants who completed T1, 932 (58.5%) also completed T2. Of these participants, 679 (72.9%) completed T3 and were included in the three-wave predictor analyses. Thus, attrition was 41.5% from T1 to T2 and 27.1% from T2 to T3 among participants who had completed the preceding wave. Attrition analyses compared the 679 three-wave completers with the remaining T1 respondents. The two groups did not differ in baseline SVA, unintentional procrastination, or self-concept clarity, all *p*s ≥ 0.607. In a multivariable retention model, baseline SVA, unintentional procrastination, and self-concept clarity were not associated with three-wave retention. Female students showed higher odds of retention than male students, OR = 1.42, 95% CI [1.06, 1.90], *p* = 0.020. All participants provided informed consent, and the study was approved by the institutional review board of the authors’ university.

### 2.2. Measures

Short Video Addiction. SVA was assessed using the Short Video Addiction Scale for College Students developed by Qin et al. (2019). The 14-item scale measures withdrawal, escape, loss of control, and inefficiency related to short video use. Sample items include “If I have not watched short videos for a while, I become anxious” and “Watching short videos directly reduces my efficiency in completing tasks.” Items were rated on a 5-point scale from 1 (completely inconsistent) to 5 (completely consistent), with higher scores indicating greater SVA risk. Internal consistency was excellent across the three waves, with Cronbach’s α = 0.940 at T1, α = 0.955 at T2, and α = 0.951 at T3.

Unintentional Procrastination. Unintentional procrastination was measured using the Unintentional Procrastination Scale (UPS; Fernie et al., 2017). The scale includes six items assessing the tendency to delay tasks despite intending to complete them. The original scale showed a unidimensional structure and evidence of construct and divergent validity, with good model fit, CFI = 0.99, TLI = 0.99, and RMSEA = 0.038 (Fernie et al., 2017). The UPS has also been used in research with Chinese adolescents (Liu et al., 2024). Sample items include “I often procrastinate, even when I intend to start tasks earlier” and “I really want to complete tasks on time, but I often fail to do so.” Items were rated on a 4-point scale from 1 (do not agree) to 4 (agree very much), with higher scores indicating higher unintentional procrastination. Internal consistency was excellent across waves, with Cronbach’s α = 0.949 at T1, α = 0.961 at T2, and α = 0.964 at T3. The longitudinal CFA results for the UPS are reported in the Section 3.1.

Self-Concept Clarity. Self-concept clarity was assessed using the Self-Concept Clarity Scale (SCCS; Campbell et al., 1996). The scale measures the clarity, consistency, and stability of self-beliefs. A Chinese 12-item adaptation has been used in Chinese adolescent samples (Niu et al., 2016). In a large Chinese sample that included university students, the same 12-item version showed satisfactory structural validity, CFI = 0.95, TLI = 0.93, RMSEA = 0.05, and SRMR = 0.03 (Yang et al., 2025). Sample items include “My beliefs about myself often conflict with one another” and “In general, I have a clear sense of who I am and what I am like.” Items were rated on a 5-point scale from 1 (completely inconsistent) to 5 (completely consistent). Following standard scoring procedures, all items except Items 6 and 11 were reverse-coded, such that higher scores indicated greater self-concept clarity. Internal consistency was good across waves, with Cronbach’s α = 0.847 at T1, α = 0.845 at T2, and α = 0.840 at T3. The longitudinal CFA results for the SCCS are reported in the Section 3.1.

### 2.3. Procedure

Data were collected by the research team across three waves, with approximately six months between adjacent waves. Surveys were administered in classroom settings, and participation was voluntary. To reduce potential pressure to participate, course instructors were not present during data collection. Participants were informed of the study purpose, the voluntary nature of participation, and the confidentiality of their responses before providing informed consent. After completion of the final wave, the research team provided the participating university with a brief summary report and delivered a feedback talk on students’ short video use, unintentional procrastination, and self-concept clarity.

### 2.4. Data Analysis

Data cleaning, record linkage, descriptive analyses, and attrition analyses were conducted in SPSS 29.0. Longitudinal CFA was conducted in R using the lavaan package, whereas latent profile and transition analyses were conducted in Mplus 8.3. The primary LTA sample comprised students with valid SVA data at two or more waves. Missing wave-specific SVA data within this analytic sample were retained and handled using full-information maximum likelihood under the missing at random assumption.

Before profile modeling, longitudinal CFA was conducted for the 14 SVA items using the original four-factor structure of the scale. Configural, metric, and scalar invariance models were compared across the three waves. The four original SVA dimensions, namely loss of control, withdrawal, escape, and inefficiency, were subsequently used as theory-based indicators in the profile analyses. Longitudinal CFA was also conducted for unintentional procrastination and self-concept clarity among participants with available item-level data across all three waves (*n* = 679). For the UPS, a one-factor model with correlated residuals for the same items across waves was evaluated. For the SCCS, a one-factor model with an orthogonal wording factor for reverse-keyed items was evaluated to account for potential method effects associated with reverse wording. Configural, metric, and scalar invariance models were compared across waves.

Separate one- through five-profile solutions were estimated at each wave within the primary LTA sample, using participants with valid SVA data at the respective wave, to examine whether a comparable number of profiles was supported across T1, T2, and T3. Model selection considered the Akaike information criterion, Bayesian information criterion, sample-size-adjusted Bayesian information criterion, entropy, the Lo–Mendell–Rubin likelihood ratio test, the bootstrap likelihood ratio test, profile size, replication of the best log-likelihood value, parsimony, and substantive interpretability. The bootstrap likelihood ratio tests were evaluated using 5000 bootstrap replications.

A three-wave LTA was then estimated using the retained profile solution. Profile comparability was examined by comparing a configural model, in which profile-specific dimension means were freely estimated across waves, with a structural similarity model that constrained corresponding profile-specific means to equality across waves. Transition probabilities were freely estimated for the T1 to T2 and T2 to T3 intervals. The retained model was used to estimate model-based transition probabilities and their 95% confidence intervals. Entropy and average posterior probabilities were reported to describe classification quality. The resulting lower, moderate, and higher SVA profiles were interpreted as relative latent profiles within the present sample and not as diagnostic categories.

An item-level invariant LTA using all 14 SVA items was estimated as a sensitivity analysis to examine whether the retained profile structure and general transition pattern were robust to an alternative indicator specification.

Primary predictor analyses focused on T1 to T3 transitions among the 679 participants who completed all three waves and had complete data on baseline unintentional procrastination, self-concept clarity, and gender. A three-step LTA procedure was implemented manually to examine prospective associations of baseline unintentional procrastination and self-concept clarity with profile transitions while accounting for classification error (Asparouhov & Muthén, 2014; Vermunt, 2010). In the first step, invariant T1 and T3 profile measurement models were estimated without covariates. In the second step, classification-error parameters derived from the measurement model were fixed. In the third step, baseline unintentional procrastination and self-concept clarity were standardized, and these variables and gender were entered as covariates. This procedure preserved the profile measurement model estimated before covariate inclusion and did not treat most likely profile membership as an error-free observed outcome. Covariate associations were estimated separately within each T1 SVA profile, with the higher SVA profile at T3 serving as the reference outcome. Odds ratios, 95% confidence intervals, and two-tailed *p*-values were reported. Sample adequacy was evaluated with reference to profile sizes, classification quality, successful replication of the best log-likelihood, and the precision of parameter estimates as reflected in their 95% confidence intervals.

## 3. Results

### 3.1. Longitudinal Measurement Invariance

The longitudinal CFA supported scalar invariance of the original four-factor SVA structure across the three waves. The configural model showed CFI = 0.917, TLI = 0.900, RMSEA = 0.043, and SRMR = 0.050. The metric model showed CFI = 0.916, TLI = 0.901, RMSEA = 0.043, and SRMR = 0.051. The scalar model showed CFI = 0.914, TLI = 0.901, RMSEA = 0.043, and SRMR = 0.051. Changes in CFI were 0.001 from the configural to the metric model and 0.002 from the metric to the scalar model.

Longitudinal CFA also supported scalar invariance for the UPS, with CFI = 0.949, TLI = 0.943, RMSEA = 0.064, and SRMR = 0.062. Changes in CFI were 0.003 from the configural to metric model and 0.002 from the metric to scalar model. For the SCCS, the model including a reverse-worded factor supported scalar invariance, with CFI = 0.955, TLI = 0.951, RMSEA = 0.040, and SRMR = 0.044. The corresponding changes in CFI were 0.000 and 0.002.

### 3.2. Identification of Relative SVA Profiles

Wave-specific comparisons of one through five profile solutions within the primary LTA sample are presented in Table 1. Across T1, T2, and T3, the three-profile solution showed acceptable classification quality, with entropy values of 0.918, 0.906, and 0.910, respectively. Information criteria decreased as additional profiles were added, and all BLRT comparisons were significant, *p*s < 0.001. However, the four- and five-profile solutions included small profiles at multiple waves, ranging from 1.29% to 4.31%. The three-profile solution was retained for the longitudinal analysis because it was consistently identified across waves and showed adequate profile sizes.

The configural and structural-similarity LTA models are compared in Table 2. The structural-similarity model showed a lower BIC and was retained for the transition analyses. The final model had an entropy of 0.802. Average posterior probabilities for the lower, moderate, and higher SVA profiles were 0.870, 0.838, and 0.909 at T1; 0.976, 0.927, and 0.968 at T2; and 0.905, 0.855, and 0.864 at T3, respectively.

Figure 1 presents the estimated means of the four SVA dimensions in the retained model. The lower SVA profile showed low scores across loss of control, withdrawal, escape, and inefficiency. The moderate SVA profile showed intermediate scores across dimensions, with relatively higher escape scores. The higher SVA profile showed consistently elevated scores across all four dimensions. These labels indicate relative latent profiles within the present sample and do not represent diagnostic categories.

As a sensitivity analysis, an invariant LTA using all 14 individual SVA items recovered an ordered three-profile structure. The item-level model converged with a replicated best log-likelihood and an entropy of 0.841. Its within-profile stability estimates were 0.515, 0.456, and 0.641 for the lower, moderate, and higher SVA profiles from T1 to T2, respectively. From T2 to T3, the corresponding estimates were 0.626, 0.508, and 0.584. These estimates were comparable to those obtained in the four-dimensional primary LTA, indicating that the overall profile structure and transition pattern were not dependent on the indicator specification.

Model-estimated profile proportions are presented in Table 3. The higher SVA profile decreased from 53.05% at T1 to 43.55% at T3. The lower SVA profile increased from 19.53% to 28.53%, whereas the moderate SVA profile remained relatively stable across waves.

### 3.3. Transitions Among Relative SVA Profiles

Model-estimated transition probabilities indicated both stability and movement among the relative SVA profiles across adjacent waves (Figure 2; Table 4). From T1 to T2, the higher SVA profile showed the greatest stability, with 66.9% of students estimated to remain in the higher profile, 95% CI [62.4%, 71.4%]. Stability estimates were 53.1% for the lower SVA profile, 95% CI [45.4%, 60.7%], and 42.9% for the moderate SVA profile, 95% CI [35.7%, 50.1%].

From T2 to T3, stability was 64.5% for the lower SVA profile, 95% CI [57.2%, 71.9%], 48.8% for the moderate SVA profile, 95% CI [41.1%, 56.5%], and 61.1% for the higher SVA profile, 95% CI [56.2%, 66.1%]. Thus, the moderate SVA profile showed the lowest within-profile stability across both intervals.

Movement occurred in both directions. From T1 to T2, 21.1% of students in the moderate SVA profile transitioned to the lower profile, whereas 36.0% transitioned to the higher profile. From T2 to T3, the corresponding probabilities were 19.2% and 32.0%. Students in the lower profile also showed some movement to the higher profile, with estimated probabilities of 27.4% from T1 to T2 and 17.6% from T2 to T3. Conversely, students in the higher profile had estimated probabilities of moving to the lower profile of 11.7% and 17.6% across the two intervals, respectively. These model-based estimates indicate that relative SVA profile membership showed both continuity and change over time.

### 3.4. Associations of Unintentional Procrastination and Self-Concept Clarity with T1 to T3 SVA Profile Transitions

A manual three-step latent transition analysis examined the prospective associations of T1 unintentional procrastination and self-concept clarity with T3 SVA profile membership among the 679 students who completed all three waves. The analysis accounted for classification uncertainty and included gender as a covariate. Associations were estimated separately within each T1 SVA profile, with the higher SVA profile at T3 serving as the reference outcome.

Among students in the moderate SVA profile at T1, higher unintentional procrastination was associated with lower odds of membership in the lower SVA profile at T3, OR = 0.234, 95% CI [0.104, 0.527], *p* < 0.001. Unintentional procrastination was not significantly associated with T3 profile membership among students in the lower or higher SVA profiles at T1.

Among students in the lower SVA profile at T1, higher self-concept clarity was associated with greater odds of membership in the lower SVA profile at T3, OR = 1.931, 95% CI [1.079, 3.457], *p* = 0.027. Among students in the moderate SVA profile at T1, higher self-concept clarity was associated with greater odds of membership in the moderate SVA profile at T3, OR = 1.740, 95% CI [1.071, 2.825], *p* = 0.025. No other associations between the predictors and T3 profile membership were statistically significant (Table 5).

Because the predictors were standardized, the odds ratios represent the change associated with a one standard deviation increase in each predictor. Among students initially in the moderate SVA profile, a one standard deviation increase in unintentional procrastination was associated with 76.6% lower odds of membership in the lower profile at T3. Among students initially in the lower SVA profile, a one standard deviation increase in self-concept clarity was associated with 93.1% greater odds of membership in the lower profile at T3. Among students initially in the moderate SVA profile, the corresponding increase in self-concept clarity was associated with 74.0% greater odds of membership in the moderate profile at T3. These estimates apply to the stated comparisons with the higher SVA profile at T3 as the reference outcome.

## 4. Discussion

This study examined relative SVA profiles and their transitions across three waves among university students. The findings identified lower, moderate, and higher SVA profiles, showed that students moved toward both lower and higher profiles over time, and indicated that unintentional procrastination and self-concept clarity were prospectively associated with specific profile comparisons between T1 and T3. These findings provided partial support for H1 and support for H2. Overall, these findings suggest that SVA can be examined through both symptom levels and changes in relative profile membership over time.

The three-profile structure likely reflects a level-based differentiation of SVA severity. Students in the lower SVA profile showed relatively low scores across loss of control, withdrawal, escape, and inefficiency, whereas students in the higher SVA profile showed consistently elevated scores across these four dimensions. The moderate profile showed intermediate scores, with relatively higher escape scores. This ordered pattern is consistent with recent profile studies showing heterogeneity in SVA symptoms among college students (Ding et al., 2024; L. Li et al., 2024; Pan et al., 2024; S. Wang & Liu, 2026). At the same time, because the higher SVA profile included a large proportion of the sample, these labels should be interpreted as relative SVA profiles within the present sample. The higher SVA profile reflected comparatively higher levels of SVA symptoms and did not indicate a clinical diagnosis of addiction.

The transition findings showed that relative SVA profile membership was not fixed across waves. This pattern may reflect the changing nature of university students’ daily routines and self-management demands. Across semesters, students may experience changes in academic workload, sleep routines, emotional stress, peer contexts, and availability of discretionary time. These changes may be associated with how often students turn to short videos for entertainment, escape, or temporary relief, and whether short video use begins to interfere with planned activities. As a result, some students may move toward lower SVA profiles, whereas others may move into higher SVA profiles. The moderate SVA profile showed considerable movement across waves, suggesting that it may represent an intermediate or less stable relative SVA state. This dynamic pattern is consistent with recent longitudinal studies showing that SVA is associated with psychological functioning over time (Qu et al., 2024; Zhang et al., 2024, 2025). However, the contextual conditions proposed here were not directly measured and should be regarded as possible theoretical explanations for the observed transitions.

Movement toward a lower SVA profile should be interpreted cautiously. Because movement was inferred from repeated self-report measurements, some apparent downward movement may reflect regression to the mean or variation specific to a particular assessment, especially among students with relatively elevated scores at an earlier wave. The longitudinal measurement-invariance analyses support the comparability of the SVA measure across waves, but they cannot eliminate these alternative explanations. Downward movement therefore indicates a change in relative latent profile membership and does not establish a persistent reduction in problematic short video use.

The association involving unintentional procrastination was observed specifically among students in the moderate SVA profile at T1. Within this group, higher unintentional procrastination was associated with lower odds of membership in the lower SVA profile at T3, with the higher SVA profile serving as the reference outcome. One possible explanation is that unintentional procrastination reflects difficulty translating intentions into action (Fernie et al., 2017). Students may recognize the need to reduce short video use but have difficulty implementing this intention consistently when short videos remain easily accessible and rewarding. This explanation is consistent with procrastination as a self-regulatory difficulty (Steel, 2007) and with studies linking problematic short video use to procrastination-related difficulties (Xie et al., 2023; Zhao & Kou, 2024). However, the process of translating intentions into action was not directly measured in this study. Unintentional procrastination was not significantly associated with T3 profile membership among students initially in the lower or higher profiles, indicating that its prospective association was specific to the moderate baseline profile in the present sample. Thus, H1 was supported among students initially in the moderate SVA profile but not among those initially in the higher profile and was therefore only partially supported.

Self-concept clarity showed a different pattern of prospective associations. Among students initially in the lower SVA profile, higher self-concept clarity was associated with greater odds of membership in the lower profile at T3, with the higher profile serving as the reference outcome. Among students initially in the moderate profile, higher self-concept clarity was associated with greater odds of membership in the moderate profile at T3. One possible explanation is that clear and stable self-beliefs provide internal standards that support the maintenance of behavioral boundaries when students encounter the immediate rewards of short video use. This interpretation is consistent with the definition of self-concept clarity as clear, internally consistent, and stable self-beliefs (Campbell et al., 1996), with evidence linking lower self-concept clarity to weaker self-control through reduced global self-continuity (Jiang et al., 2023), and with longitudinal evidence showing a prospective association between higher self-concept clarity and lower subsequent SVA severity (Zhang et al., 2025). However, the present study did not directly test whether self-concept clarity operated through self-guiding standards or behavioral boundaries. These processes should therefore be regarded as theoretical explanations for the observed associations. Because higher self-concept clarity was associated with a lower relative likelihood of higher-profile membership in both baseline groups specified in the hypothesis, H2 was supported.

Together, these findings suggest that the prospective associations varied according to students’ baseline profiles and the T3 profiles being compared. The finding for unintentional procrastination may be consistent with difficulty carrying out intentions to reduce an established pattern of use, whereas the findings for self-concept clarity may be consistent with maintaining existing behavioral standards. These explanations are consistent with the observed associations, but the underlying psychological processes were not directly tested.

### 4.1. Implications

Theoretically, this study shifts the focus of SVA research from SVA severity at a single assessment to changes in relative profile membership over time. Existing studies mainly explain why some students report higher SVA severity than others, but they provide less insight into why students remain in the same relative profile or move toward lower or higher SVA profiles over time (Qu et al., 2024; Zhang et al., 2024, 2025). By identifying transitions among lower, moderate, and higher SVA profiles, the present study suggests that theories of SVA need to explain both profile stability and movement over time. This distinction matters because the relevance of characteristics related to self-regulation may depend on students’ initial profiles and the direction of their subsequent movement. Among students initially in the moderate SVA profile, the association between unintentional procrastination and a lower likelihood of subsequent lower profile membership is consistent with difficulty translating intentions into action as one possible explanation for limited downward movement. The associations involving self-concept clarity are consistent with the possibility that clear self-guiding standards help students maintain lower or moderate SVA profile membership. Thus, the study extends self-regulation accounts of SVA by showing that different characteristics related to self-regulation may be relevant to different profile transitions. These interpretations remain theoretical because the underlying psychological processes were not directly tested.

This theoretical distinction also has practical implications. If the findings are replicated, information about students’ relative SVA profiles and characteristics related to self-regulation may help inform more differentiated forms of support. Self-concept clarity may be a relevant consideration when supporting students in the lower or moderate SVA profiles, with goal clarification, value reflection, routine building, and behavioral boundary setting representing potential considerations for support design. For students in the moderate SVA profile who report greater unintentional procrastination, implementation intentions, time blocking, cue control, app use boundaries, and planned interruption strategies may also warrant consideration. These suggestions are based on the observed prospective associations and should not be interpreted as evidence that these strategies are effective in changing SVA profile membership.

### 4.2. Limitations and Future Studies

Several limitations should be noted. First, the sample was drawn from a single university and was predominantly female. This composition limits the generalizability of the findings, particularly to male students, students from other institutions, and populations from different educational and cultural contexts. Future studies should examine whether the identified profiles, transition patterns, and predictor associations can be replicated in more diverse and gender balanced samples (Henrich et al., 2010; Jager et al., 2017). Second, response availability declined across waves. Although attrition analyses did not indicate systematic differences in the measured baseline variables, missingness may still have been related to unmeasured characteristics. The findings should therefore be replicated in samples with stronger follow-up retention. Third, the lower, moderate, and higher SVA profiles were primarily differentiated by average levels of SVA symptoms across the four original dimensions. They should be interpreted as relative risk patterns within this sample, not as diagnostic categories or necessarily as qualitatively distinct symptom configurations. Because the profiles mainly captured quantitative differences in SVA levels, future person-centered research should incorporate indicators from other domains, including emotional functioning, sleep, academic functioning, and social context, to identify qualitatively distinct patterns and examine cross-domain configurations involving SVA. Fourth, the estimated associations with profile transitions depended on the retained profile solution, and several transition-specific estimates had relatively wide confidence intervals, particularly for comparisons involving smaller baseline profiles. Larger longitudinal samples could provide more precise estimates of transition-specific associations and permit examination of a broader range of potential predictors. Fifth, SVA, unintentional procrastination, and self-concept clarity were assessed using self-report measures administered in classroom settings. Shared method variance and social desirability bias may have influenced the observed associations. Future studies could combine self-report data with behavioral indicators of short video use, device-based records, or informant reports. Finally, the findings should be interpreted as prospective associations. The observational design cannot rule out unmeasured confounding or reciprocal associations. Experimental, intervention, and intensive longitudinal designs are needed to clarify how changes in characteristics related to self-regulation and SVA risk unfold over time. In particular, future intervention research should examine whether strengthening self-concept clarity or addressing difficulties in translating intentions into action is followed by changes in SVA profile membership. Such research could directly evaluate the possible support strategies identified above and determine whether their effects differ according to students’ initial SVA profiles.

## Figures and Tables

**Figure 1:**
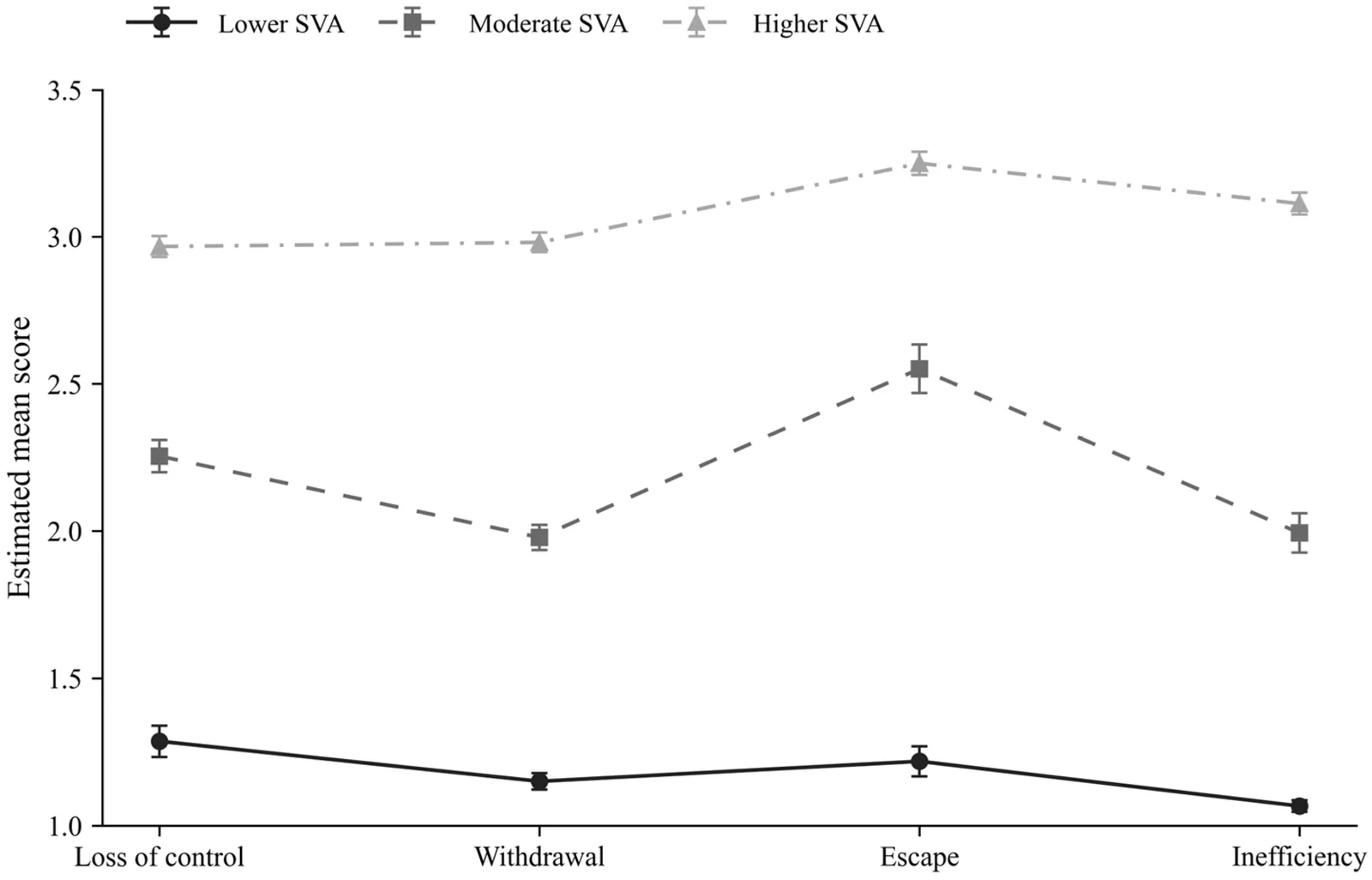
Estimated means of the four SVA dimensions across relative SVA profiles. Note. Error bars represent 95% confidence intervals. Profile-specific means were constrained to be equal across T1, T2, and T3 in the retained structural-similarity model.

**Figure 2:**
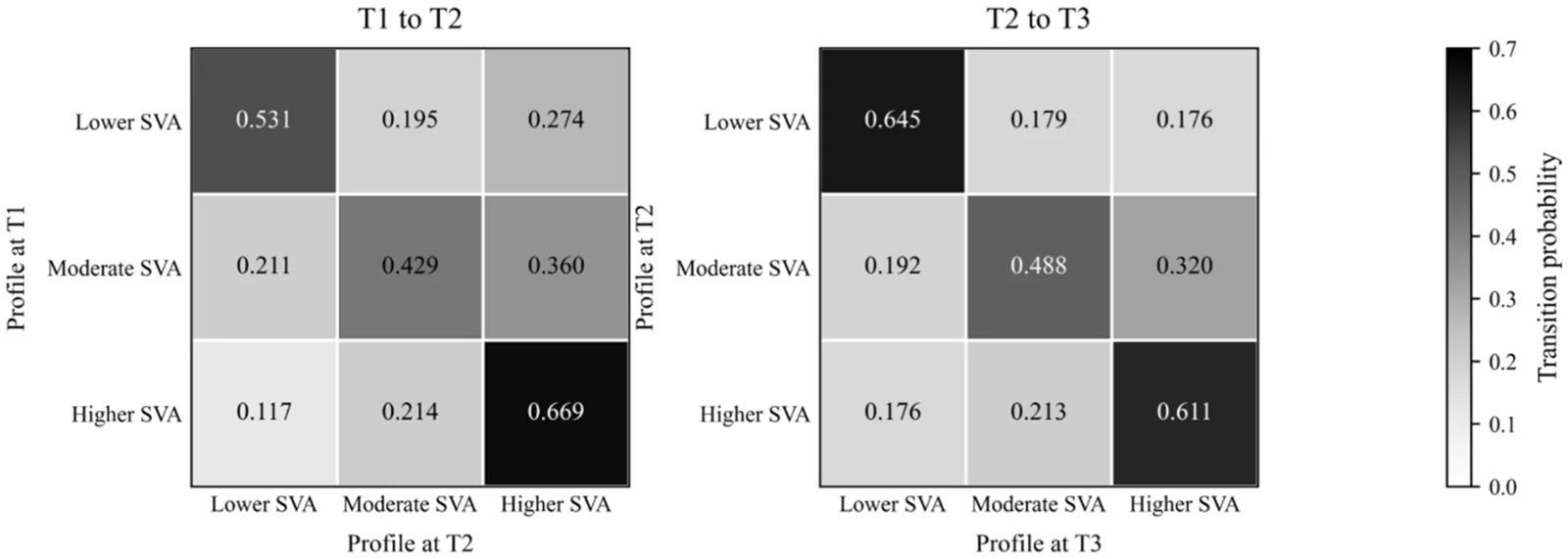
Model-estimated transition probabilities among relative SVA profiles. Note. Values represent model-estimated transition probabilities. Rows indicate profile membership at T1 or T2, and columns indicate profile membership at T2 or T3. Darker shading indicates a higher transition probability. The profiles are relative latent profiles within the present sample and not diagnostic categories.

**Table 1:** Fit indices for solutions with one to five profiles at each wave.

Wave	N	Profiles	AIC	BIC	aBIC	Entropy	LMR-LRT *p*	BLRT *p*	Smallest Profile, *n* (%)
T1	933	1	10,166.24	10,204.95	10,179.54	-	-	-	933 (100.00)
		2	8304.71	8367.61	8326.32	0.891	<0.001	<0.001	350 (37.51)
		3	7790.05	7877.15	7819.98	0.918	<0.001	<0.001	60 (6.43)
		4	7224.01	7335.29	7262.24	0.926	0.038	<0.001	48 (5.15)
		5	7083.20	7218.68	7129.75	0.936	<0.001	<0.001	12 (1.29)
T2	1134	1	12,125.47	12,165.74	12,140.33	-	-	-	1134 (100.00)
		2	9482.94	9548.38	9507.09	0.914	<0.001	<0.001	383 (33.77)
		3	8802.21	8892.81	8835.64	0.906	<0.001	<0.001	254 (22.40)
		4	8315.77	8431.54	8358.49	0.937	<0.001	<0.001	33 (2.91)
		5	8143.51	8284.45	8195.52	0.929	0.258	<0.001	33 (2.91)
T3	882	1	9450.52	9488.77	9463.37	-	-	-	882 (100.00)
		2	7392.90	7455.07	7413.79	0.898	<0.001	<0.001	395 (44.79)
		3	6820.07	6906.15	6848.98	0.910	<0.001	<0.001	238 (26.98)
		4	6568.02	6678.01	6604.96	0.938	<0.001	<0.001	26 (2.95)
		5	6320.77	6454.67	6365.75	0.960	0.404	<0.001	38 (4.31)

Note. AIC = Akaike information criterion; BIC = Bayesian information criterion; aBIC = sample-size-adjusted Bayesian information criterion; LMR-LRT = Lo–Mendell–Rubin likelihood ratio test; BLRT = bootstrap likelihood ratio test. All BLRT comparisons were based on 5000 successful bootstrap draws. Smallest profile sizes are based on most likely profile membership.

**Table 2:** Profile comparability model comparison.

Model	Free Parameters	Log Likelihood	AIC	BIC	aBIC	Entropy
Configural model	62	−11,499.61	23,123.21	23,435.34	23,238.41	0.820
Structural similarity model	38	−11,570.68	23,217.36	23,408.67	23,287.97	0.802

Note. The retained structural-similarity model constrained corresponding profile-specific means to equality across T1, T2, and T3. Transition probabilities were freely estimated for the T1 to T2 and T2 to T3 intervals. AIC = Akaike information criterion; BIC = Bayesian information criterion; aBIC = sample-size-adjusted Bayesian information criterion.

**Table 3:** Model-estimated relative SVA profile proportions across the three waves.

Wave	Observed SVA N	Lower SVA	Moderate SVA	Higher SVA
T1	933	19.53%	27.43%	53.05%
T2	1134	22.36%	26.92%	50.72%
T3	882	28.53%	27.92%	43.55%

Note. Profile proportions are model-estimated from the retained structural-similarity LTA. The LTA analytic sample included 1135 participants with valid SVA data at two or more waves; all available data contributed to model estimation under full-information maximum likelihood. Observed SVA N indicates the number of participants with observed SVA data at each wave. The profile proportions are not diagnostic prevalence estimates. Model-estimated proportions from the final LTA may differ from the wave-specific profile proportions because the LTA incorporated information across waves and imposed structural-similarity constraints.

**Table 4:** Model-estimated transition probabilities across relative SVA profiles.

Earlier Profile	Lower SVA	Moderate SVA	Higher SVA
T1 to T2			
Lower SVA	0.531 [0.454, 0.607]	0.195 [0.130, 0.260]	0.274 [0.207, 0.342]
Moderate SVA	0.211 [0.155, 0.267]	0.429 [0.357, 0.501]	0.360 [0.291, 0.429]
Higher SVA	0.117 [0.088, 0.146]	0.214 [0.173, 0.255]	0.669 [0.624, 0.714]
T2 to T3			
Lower SVA	0.645 [0.572, 0.719]	0.179 [0.116, 0.243]	0.176 [0.120, 0.232]
Moderate SVA	0.192 [0.135, 0.248]	0.488 [0.411, 0.565]	0.320 [0.251, 0.389]
Higher SVA	0.176 [0.137, 0.216]	0.213 [0.168, 0.257]	0.611 [0.562, 0.661]

Note. Values are model-estimated transition probabilities with 95% confidence intervals. Rows identify the profile at T1 or T2, and columns identify the profile at T2 or T3. SVA = short video addiction. The profiles are relative latent profiles within the present sample and not diagnostic categories.

**Table 5:** Associations of unintentional procrastination and self-concept clarity with T1 to T3 SVA profile membership.

Predictor	T1 SVA Profile	Estimated T1 Profile *n*	T3 SVA Profile	b	SE	OR [95% CI]	*p*
Unintentional procrastination	Lower	130	Lower	0.307	0.366	1.359 [0.663, 2.787]	0.402
			Moderate	0.335	0.497	1.398 [0.527, 3.705]	0.501
	Moderate	188	Lower	−1.451	0.413	0.234 [0.104, 0.527]	<0.001
			Moderate	−0.350	0.305	0.705 [0.387, 1.283]	0.252
	Higher	361	Lower	−0.112	0.168	0.894 [0.643, 1.242]	0.504
			Moderate	0.023	0.183	1.023 [0.715, 1.465]	0.899
Self-concept clarity	Lower	130	Lower	0.658	0.297	1.931 [1.079, 3.457]	0.027
			Moderate	0.490	0.406	1.632 [0.737, 3.614]	0.227
	Moderate	188	Lower	0.158	0.330	1.171 [0.613, 2.236]	0.633
			Moderate	0.554	0.247	1.740 [1.071, 2.825]	0.025
	Higher	361	Lower	0.158	0.218	1.171 [0.763, 1.797]	0.469
			Moderate	0.270	0.196	1.309 [0.891, 1.925]	0.170

Note. Estimates are from the three-step model using the three-wave predictor sample (*n* = 679). Estimated T1 profile counts are rounded to the nearest whole number because baseline profile membership was modeled probabilistically. Predictors were standardized and mutually adjusted for the other predictor and gender. Within each T1 SVA profile, the higher SVA profile at T3 was the reference outcome. OR = odds ratio; CI = confidence interval.

## Data Availability

The original contributions presented in this study are included in the article. Further inquiries can be directed to the corresponding author.

## Supplementary Materials

The following supporting information can be downloaded at https://www.mdpi.com/article/10.3390/bs16101724/s1, Figure S1: Participant Flow Diagram.
