# Supplementary material for: Short Video Addiction Risk Transitions Among University Students: A Three-Wave Latent Transition Analysis of Unintentional Procrastination and Self-Concept Clarity
Source: Behav Sci (Basel). 2026 Sep 22;16(10):1724. doi: 10.3390/bs16101724 (PMC13701369; doi:10.3390/bs16101724)
Supplement: Supplementary file 1 [file behavsci-16-01724-s001.zip › behavsci-4537077-supplementary.pdf]

Supplementary Materials

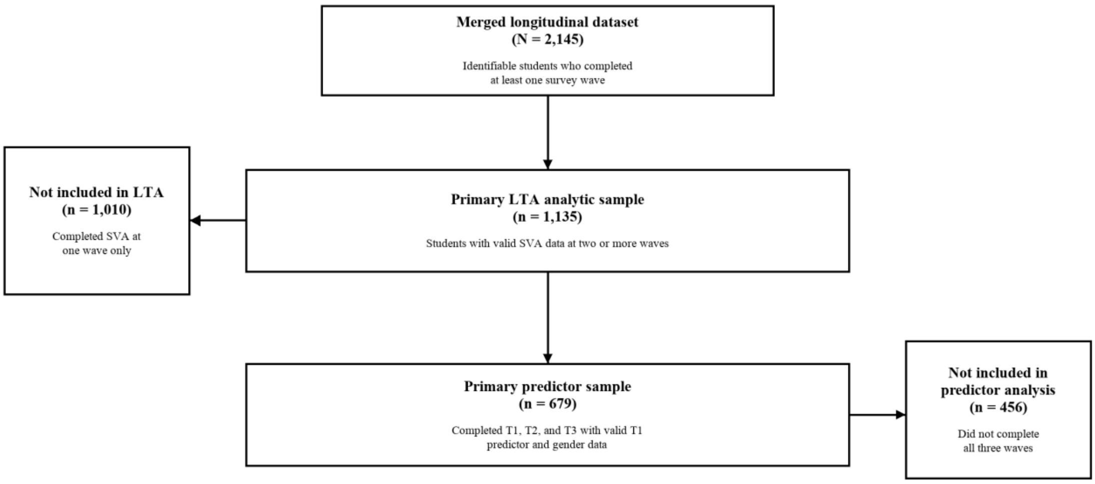

Figure S1. Participant Flow Diagram

Note. LTA = latent transition analysis; SVA = short-video addiction. The numbers of participants with available SVA data were 1,592 at T1, 1,352 at T2, and 1,015 at T3. These wave-specific counts are not mutually exclusive.
